# Supplementary material for: The effect of overnight consolidation in the perceptual learning of non-native tonal contrasts
Source: PLoS One. 2019 Dec 12;14(12):e0221498. doi: 10.1371/journal.pone.0221498 (PMC6907771; doi:10.1371/journal.pone.0221498)
Supplement: Attachment — Submitted filename: Review for PLoS One_revised1.pdf [file pone.0221498.s002.pdf]

Summary

This study explored the effects of memory consolidation after sleep on the perceptual learning of new lexical tone contrasts. Two groups of native Mandarin speakers were trained to identify 3 Cantonese level tones [T1 (high-level), T3 (mid-level) and T6 (low-level)]. One group was trained in the morning and tested immediately after training, after a 12-hour delay and after a 24-hour delay. The other group was trained in the evening and tested immediately after training, 12 hours later after a night of sleep, and again in the evening, after a 24-hour delay. Both groups were administered pitch sensitivity and pitch memory tasks and an AX perceptual discrimination task before the identification (ID) training. After training, they were administered both the AX as well as the ID posttests. The results showed a significant increase in tone identification accuracy from posttest 1 to posttest 3 for both trained and untrained talker among the evening training group but not among the morning training group. No significant difference was found between the two groups in the AX discrimination posttests. The authors concluded that "overnight consolidation facilitates generalization across talkers in the identification of novel Cantonese level-level tonal contrasts."

Comments

The manuscript is much improved and is almost ready for publication. One final suggestion I would like to make is for the authors to entertain another possible explanation for the "divergent performance changes over time in the identification tasks, but not in the discrimination tasks" between the two training groups. It has been shown (e.g., Guenther et al., 1999) that, for non-speech stimuli, a categorization training "in which subjects learn to identify stimuli within a particular frequency range as members of the same category, can lead to a decrease in sensitivity to stimuli in that category", whereas a discrimination training using the same set of stimuli lead to "an increase in sensitivity to differences" among the training stimuli. In other words, "acquired similarity" is observed in the identification training, while the opposite effect is observed in the discrimination training. Since the identification training promotes detection of features/dimensions that the two stimuli shared, then it would make sense for the two groups to differ on the identification task due to the overnight consolidation effect, but they would be comparable in a task that promotes detection of acoustic differences for which they were not trained. This account is similar, but not identical to the 'explicit recall of tonal category' vs 'implicit ability to detect acoustic differences' explanation that the authors have already proposed.

Reference

Guenther, F. H., Husain, F. T., Cohen, M. A., & Shinn-Cunningham, B. G. (1999). Effects of categorization and discrimination training on auditory perceptual space. *The Journal of the Acoustical Society of America*, *106*(5), 2900-2912.
