# Supplementary material for: The effect of overnight consolidation in the perceptual learning of non-native tonal contrasts
Source: PLoS One. 2019 Dec 12;14(12):e0221498. doi: 10.1371/journal.pone.0221498 (PMC6907771; doi:10.1371/journal.pone.0221498)
Supplement: Attachment — Submitted filename: Response to Reviewers.docx [file pone.0221498.s003.docx]

Dear Dr. Mulak,

We would like to thank you for giving us the opportunity to revise our manuscript, and the Reviewer 2 for her insightful comment regarding the identification and discrimination training/tasks. We have added the alternative explanation suggested by Reviewer 2. Below, we elaborated on how we have addressed her comment. We also referred to specific changes of the marked-up copy (e.g., page number), highlighted in red.

We hope that you will find the revised manuscript satisfactory, and look forward to hearing back from you soon.

Sincerely,

Zhen Qin, Caicai Zhang

**Re: comments of Reviewer 2**

1. *One final suggestion I would like to make is for the authors to entertain another possible explanation for the “divergent performance changes over time in the identification tasks, but not in the discrimination tasks” between the two training groups. It has been shown (e.g., Guenther et al., 1999) that, for non-speech stimuli, a categorization training “in which subjects learn to identify stimuli within a particular frequency range as members of the same category, can lead to a decrease in sensitivity to stimuli in that category”, whereas a discrimination training using the same set of stimuli lead to “an increase in sensitivity to differences” among the training stimuli. In other words, “acquired similarity” is observed in the identification training, while the opposite effect is observed in the discrimination training. Since the identification training promotes detection of features/dimensions that the two stimuli shared, then it would make sense for the two groups to differ on the identification task due to the overnight consolidation effect, but they would be comparable in a task that promotes detection of acoustic differences for which they were not trained. This account is similar, but not identical to the ‘explicit recall of tonal category’ vs ‘implicit ability to detect acoustic differences’ explanation that the authors have already proposed.*

- We followed Reviewer 2’s suggestion, and added another possible explanation accounting for the “divergent performance changes over time in the identification tasks, but not in the discrimination tasks” between the two training groups (p. 32-33).
- Specifically, as suggested, the different underlying processes of the identification and discrimination training involving non-speech stimuli were discussed. References (Wayland & Li, 2008) on the effect of the training methods on non-native tones were also added. In addition, the effect of our identification training on the two training groups’ performance in the tone identification and discrimination task was elaborated in terms of the training/task nature.

Reference:

Wayland, R. P., & Li, B. (2008). Effects of two training procedures in cross-language perception of tones. *Journal of Phonetics*. https://doi.org/10.1016/j.wocn.2007.06.004
